# SARS-CoV-2 vaccines: a triumph of science and collaboration

**DOI:** 10.1172/jci.insight.149187

**Published:** 2021-05-10

**Authors:** Jonathan L. Golob, Njira Lugogo, Adam S. Lauring, Anna S. Lok

**Affiliations:** 1 Division of Infectious Diseases,; 2 Division of Pulmonary and Critical Care Medicine, and; 3 Division of Gastroenterology and Hepatology, Department of Internal Medicine, University of Michigan Medical School, Ann Arbor, Michigan, USA.

## Abstract

Roughly 1 year after the first case of COVID-19 was identified and less than 1 year after the sequencing of SARS-CoV-2, multiple SARS-CoV-2 vaccines with demonstrated safety and efficacy in phase III clinical trials are available. The most promising vaccines have targeted the surface glycoprotein (S-protein) of SARS-CoV-2 and achieved an approximate 85%–95% reduction in the risk of symptomatic COVID-19, while retaining excellent safety profiles and modest side effects in the phase III clinical trials. The mRNA, replication-incompetent viral vector, and protein subunit vaccine technologies have all been successfully employed. Some novel SARS-CoV-2 variants evade but do not appear to fully overcome the potent immunity induced by these vaccines. Emerging real-world effectiveness data add evidence for protection from severe COVID-19. This is an impressive first demonstration of the effectiveness of the mRNA vaccine and vector vaccine platforms. The success of SARS-CoV-2 vaccine development should be credited to open science, industry partnerships, harmonization of clinical trials, and the altruism of study participants. The manufacturing and distribution of the emergency use–authorized SARS-CoV-2 vaccines are ongoing challenges. What remains now is to ensure broad and equitable global vaccination against COVID-19.

## Introduction

The COVID-19 pandemic, caused by the novel coronavirus SARS-CoV-2, has caused massive global upheaval. The risk of death is higher in elderly individuals, Black people, Hispanic people, people who are immunocompromised, and those with medical comorbidities. Even in those who survive SARS-CoV-2 infection, a substantial percentage suffer persistent and debilitating symptoms (1, 2). While some communities have been able to contain the pandemic with a combination of public health measures (including extensive testing followed by isolation and tracing of contacts, near-universal masking, and targeted quarantines), most communities have failed to halt the pandemic. Much hope now resides in the potential of SARS-CoV-2 vaccines to reduce the risk of disease and infection.

The open exchange of data on the SARS-CoV-2 genome sequence and the collaboration and co-investment of governments, the pharmaceutical industry, and academic laboratories have led to the rapid development of multiple highly promising SARS-CoV-2 vaccines. As of March 22, 2021, 82 vaccines are in clinical development, including 22 in phase III or II/III trials (3). Roughly 1 year after the first case of COVID-19 was identified, three vaccines received emergency use authorization (EUA) by the US FDA, and a fourth vaccine received EUA by the European Medicines Agency (EMA). The novel technologies used to develop these vaccines also contributed to the speed with which candidate vaccines were developed.

Here, we review immunity to SARS-CoV-2 infection, the different vaccine technologies in use, the most-promising vaccine candidates, and the current evidence for safety and efficacy. We also review the ongoing challenges of meeting vaccination goals, including production and supply chain issues, timely and equitable distribution, vaccine hesitancy, and the constant threat of emerging virus variants.

## SARS-CoV-2 immunity after natural infection

The immunological correlates of protection against SARS-CoV-2 are largely unknown. Virus-specific antibodies (IgA and IgM followed by IgG) against viral surface glycoproteins, mainly the spike (S) glycoprotein, are detected within 7 to 10 days after illness onset. Some studies suggest that neutralizing antibodies are short lived, falling to undetectable levels within a few months. However, a study from Iceland found that 91% of infected individuals remained seropositive after 4 months (4). In addition, virus-specific T cell immunity is activated in parallel, with a cytotoxic phenotype during acute infection and a memory phenotype during the convalescent phase (5, 6). Of note, T cell responses have been detected in patients who have recovered from COVID-19 with no detectable antibodies. Thus, while antibody titers may decline over time, it is possible that immunity may persist. A study of 12,541 health care workers at the Oxford University Hospitals showed the presence of anti-S or anti-nucleocapsid antibodies (including high “negative” titers, i.e., detected but below quantification) was associated with a substantially reduced risk of RT-PCR–confirmed SARS-CoV-2 infection over 31 weeks of follow-up screening (7). Rare cases of reinfection have been documented, suggesting that immunity after natural infection may not be sterilizing but reinfection cases tended to be mild (7). Additional data are needed to confirm whether natural immunity can ameliorate disease severity and shorten viral shedding in those who are reinfected, the latter of which is key for reducing transmission.

It is likely that the immune response after vaccination will be distinct compared with that after natural infection. Accessory proteins within SARS-CoV-2 disrupt the activation of IFNs (8–10), which are in turn crucial for organizing an optimal immune response to viral infections (11). This has led to the concept of an “imbalanced” immune response to natural SARS-CoV-2 infection (8), with inappropriate inflammation and cellular responses contributing to the pathogenesis of severe COVID-19. The accessory proteins of SARS-CoV-2 are not present in the vaccines, leading to hope of a superior immune response to vaccines as compared with natural infection with SARS-CoV-2.

## Harmonization of SARS-CoV-2 vaccine efforts

Vaccines have traditionally taken more than 10 years from identification of targets and selection of platforms to completion of phase I, II, and III clinical trials and ultimate regulatory approval. The massive scale of SARS-CoV-2 infection worldwide and the enormous death toll called for a different approach. In May 2020, the US government established Operation Warp Speed, a public-private partnership to accelerate the development, production, and distribution of COVID-19 vaccines, therapeutics, and diagnostics. The contributing stakeholders include the CDC, the US FDA, the NIH, and the Biomedical Advanced Research and Development Authority.

Harmonization of the development of SARS-CoV-2 vaccines was the objective of Operation Warp Speed. A forum for scientific exchange and partnership was established (e.g., the NIH’s involvement in the development of the Moderna vaccine). The FDA provided input into trial design and accelerated review as results became available. The US government provided more than $18 billion toward the development and prepurchasing of hundreds of millions of doses before trial completion of candidate vaccines, reducing the financial risk of vaccine manufacturers.

Despite the urgency, development of SARS-CoV-2 vaccines proceeded through the same steps as other vaccines: target identification, platform selection, design of candidate vaccines, and phased human clinical trials. Phase I/II trials focus on dose finding, safety, and measures of immunogenicity (e.g., the development of neutralizing antibodies against SARS-CoV-2 S protein). Phase III trials needed to be double blind, randomized, and placebo controlled, and a minimum of 2 months of safety data was required for consideration for EUA. Coordination from Operation Warp Speed across different trials ensures that similar data are collected, endpoints are harmonized, and standardized assays are used to evaluate immune response. Operation Warp Speed also monitors enrollment to assure that each trial will have sufficient representation of high-risk groups: elderly individuals, Hispanic and Black people, and those with medical comorbidities. However, many important groups, such as children and adolescents, pregnant and lactating women, immunocompromised individuals, and people with cancers were not included in the initial phase III trials. Additional trials are ongoing or planned for these groups.

In phase I and II clinical trials, immunological response to COVID-19 vaccine is mainly determined based on detection of antibodies against the S protein and the receptor-binding domain (RBD). Protection against SARS-CoV-2 infection is assumed based on detection of antibody titers similar to that found in convalescent plasma and results of antibody detection by ELISA comparable to levels that are effective in virus neutralization assays. Several studies have also demonstrated the induction of cellular immune response, predominantly a T helper 1–biased CD4^+^ T cell response. In addition, some vaccines have been demonstrated to be effective in protecting against SARS-CoV-2 challenge studies in nonhuman primates (12–14).

## Assessment of SARS-CoV-2 vaccine efficacy in clinical trials

The SARS-CoV-2 vaccine phase III trials have set any case of RT-PCR–confirmed COVID-19 as the primary endpoint, because it was felt that a requirement to show efficacy in preventing severe disease or death would require enrollment of a much larger number of participants, potentially delaying the determination of vaccine efficacy. The FDA and WHO suggested that laboratory-confirmed COVID-19 or SARS-CoV-2 infection is an appropriate primary endpoint and that vaccine efficacy of at least 50% should be demonstrated in placebo-controlled trials. The three vaccines that have received EUA from the FDA used symptomatic infection, confirmed by RT-PCR testing, after completion of the course of vaccine as a primary endpoint. As expected, severe infections are rare in both placebo and vaccine arms of the phase III trials; while statistically underpowered, there is an indication the vaccines ameliorate disease severity.

Each trial was designed to follow the participants for 2 years to evaluate durability of protection and included longitudinal blood sample collection to allow for analysis of the efficacy of these vaccines in preventing asymptomatic infection, seroconversion rates, and durability of antibody response; results of these analyses are not yet available.

## SARS-CoV-2 vaccine technologies and efficacy in phase III clinical trials

**Figure 1:**
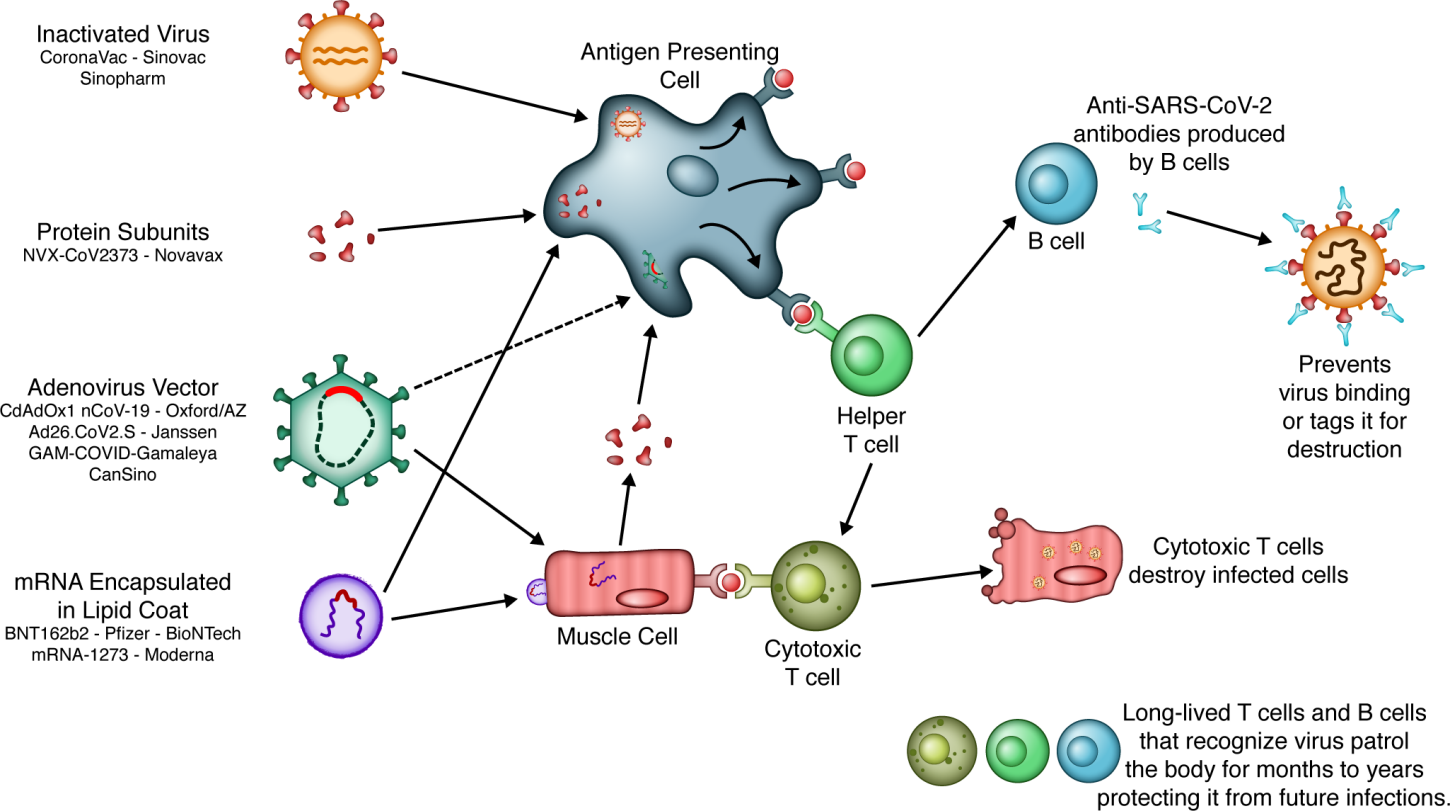
SARS-CoV-2 vaccine platforms. A schematic representation of the major SARS-CoV-2 vaccine platforms under active development as of February 2021. Adenoviral vector vaccines refer to replication-incompetent adenoviral-based vectors. All candidate vaccines (aside from whole inactivated virus) target the binding domain of the SARS-CoV-2 spike (S) protein. Both mRNA and vector vaccines target muscle cells at the site of injection. The muscle cells produce (portions of the) SARS-CoV-2 spike protein, which is in turn presented via MHC class I to antigen-presenting cells and cytotoxic T cells. In contrast, protein subunit vaccines and inactivated viral vaccines are directly taken up by antigen-presenting cells. The antigen-presenting cells in turn present the spike protein antigen to T helper cells and B cells, resulting in an orchestrated humoral and cellular immune response against the spike protein of SARS-CoV-2, including the generation of memory T cells and B cells to respond to future exposures to SARS-CoV-2. Illustrated by Rachel Davidowitz.

**Figure 2:**
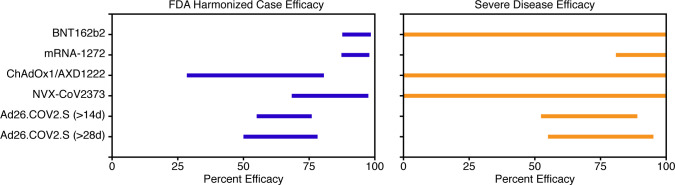
Comparison of the efficacy of COVID-19 vaccines as presented in the interim analyses of phase III clinical trials. All results are based on cases that occurred at least 2 weeks after the final vaccine dose. CIs are calculated using the Clopper and Pearson method (51) and are not adjusted for differences in the underlying study populations. The left panel shows the estimated efficacy against the FDA-harmonized case definition for symptomatic COVID-19 (any COVID-19 symptoms and a confirmation of SARS-CoV-2 infection via RT-PCR testing). The right panel shows the estimated efficacy against severe/critical COVID-19. The wide CIs for some of the vaccines are due to the small number of severe cases at the time of interim analysis. Illustrated by Rachel Davidowitz.

As of February 2021, four vaccine platforms with candidate SARS-CoV-2 vaccines have reached phase III clinical trials: mRNA, nonreplicating viral vector, protein subunit, and inactivated virus (Figure 1 and Table 1). Interim results of phase III trials from mRNA, protein subunit, and nonreplicating viral vector vaccines have been released, offering a first glimpse of the efficacy of these platforms (Figure 2 and Table 1).

### mRNA.

mRNA vaccines deliver a SARS-CoV-2 S protein–encoding mRNA template stabilized in its prefusion conformation in a lipid nanoparticle capsule. The advantage of this approach is that the SARS-CoV-2 S protein is produced by the vaccine recipients’ cells, such that the target antigens can be processed for presentation via class I and II MHC from the transfected cells and professional antigen-presenting cells, respectively. This induces protective immunity by priming antigen-specific CD4^+^ T helper and CD8^+^ cytotoxic T cell immune response as well as neutralizing antibody response from B cells. The lipid envelope serves as a delivery vehicle and the mRNA, via stimulation of TLR7 and TLR8, can also act as an adjuvant (15). Direct delivery of nucleic acids without the need for a viral vector also eliminates the risk of preexisting immunity, which may diminish efficacy (16).

The promise of mRNA vaccines was borne out by the first two SARS-CoV-2 vaccines that received FDA EUA: BNT162b2 (Pfizer/BioNTech) and mRNA-1273 (Moderna/NIH). The BNT162b2 vaccine achieved an estimated efficacy of 95.0% (95% CI, 90.3%–97.6%) against symptomatic COVID-19 7 days after the second dose (17) among participants without prior infection. The mRNA-1273 vaccine had comparable efficacy of 94.1% (95% CI, 89.3%–96.8%) 14 days after the second dose (18). Both vaccines showed similar efficacy in individuals aged 65 years or older, Hispanic people, Black people, people with obesity, and those with comorbidities that increase the risk of severe COVID-19. Across both trials, none of the vaccinated participants required hospitalization for COVID-19, and a protective effect was observed beginning 14 days after the first dose.

The mRNA technology is versatile, and new vaccines against emerging variants can be rapidly manufactured if deemed necessary.

### Nonreplicating viral vectors.

These candidate vaccines employ a viral vector, a common cold-causing adenovirus, genetically engineered so that it cannot replicate in the host, with the SARS-CoV-2 S protein–encoding sequence inserted. Similar to the mRNA approach, the viral vector approach uses the host cellular machinery for transcription of the SARS-CoV-2 S protein gene to mRNA and then translation to the SARS-CoV-2 S protein. The SARS-CoV-2 vaccines in phase III trials using this technology include the ChAdOx1 nCoV-19 (AstraZeneca/University of Oxford), the Ad26.COV2.S (Janssen), the Gam-COVID-Vac (Gamaleya/Health Ministry of the Russian Federation), and the CanSino Biologics vaccines. The immune stimulant is the viral vector itself.

A major challenge to this approach is the potential presence of preexisting cellular immunity or neutralizing antibodies against the viral vector (16). Avoiding preexisting anti-adenoviral immunity is the basis for the choice of a less common adenovirus serotype (human adenovirus type 26 in the Janssen vaccine) or a nonhuman adenovirus (chimpanzee adenovirus in the AstraZeneca vaccine). The Gam-COVID-Vac vaccine uses adenovirus type 26 as vector in the first dose and adenovirus type 5 in the second dose. The CanSino vaccine uses adenovirus type 5 as vector. Although viral vector vaccines have been studied in HIV and other diseases, only one vaccine, the Janssen Ebola vaccine, has come to fruition, receiving EUA from EMA in 2020 (19). Phase II trials of both ChAdOx1 nCoV-19 and Ad26.COV2.S vaccines have demonstrated good safety profiles and immunogenicity (20, 21) as well as protection in animal models (12, 13).

An interim analysis of the Ad26.COV2.S vaccine phase III trial served as the basis for EUA by the FDA (22). Efficacy against moderate-to-severe/critical COVID-19 was 66.9% (95% CI, 59.0%–73.4%) and 66.1% (95% CI, 55.0%–74.8%) 14 and 28 days after the single-dose vaccine. Estimated efficacy against severe/critical COVID-19 was 76.7% (95% CI, 54.6%–89.1%) at 14 days, increasing to 85.4% (95% CI, 54.2%–96.9%) by 28 days. Efficacy was overall similar for people over 60 years old. These data are impressive for a single-dose vaccine, which provides significant advantages during distribution and administration.

The efficacy of the ChAdOx1 nCoV-19 vaccine was reported from phase III trials in the United Kingdom and Brazil (23). The interpretation of the results of these trials is complicated by a dosing error (in which some participants unintentionally received a half-dose for their first of two doses), a small number of participants, and differences in efficacy in the two countries. The overall efficacy in preventing symptomatic infection more than 14 days after the second dose was 70.4% (95% CI, 54.8%–80.6%), with efficacy of 62.1% (95% CI, 41.0%–75.7%) in those who received standard doses and 90.0% (95% CI, 67.4%–97.0%) in those who received a half-dose followed by a standard dose. Notably, the lower efficacy was based on results from the study in Brazil, and higher efficacy was reported for the study in the United Kingdom. Hospitalizations and severe COVID-19 occurred rarely but exclusively in the placebo arm of these trials. Due to varying intervals between the first and second doses, vaccine efficacy after a single standard dose from day 22 to day 90 was modeled and estimated to be 76% (95% CI, 59%–86%), with maintenance of antibody levels up to day 90. Furthermore, vaccine efficacy appeared to be 82.4% (95% CI, 62.7%–91.7%) when the interval between doses was more than 12 weeks compared with 54.9% (95% CI, 32.7%–69.7%) when the interval was less than 6 weeks. Similarly, geometric mean antibody levels were higher with a longer prime-boost interval in those age 18–55 years old (24). A larger phase III trial using two standard doses 28 days apart with a majority of the participants in the US recently completed enrollment. Preliminary results of this trial showed vaccine efficacy of 76% (95% CI, 68%–82%) at preventing symptomatic infection and 100% efficacy at preventing severe or critical disease and hospitalization. Vaccine efficacy was consistent across ethnicity and age. The ChAdOx1 nCoV-19 vaccine has received EUA from the United Kingdom and the European Union.

In the United Kingdom, a single-blind multicenter randomized phase II/III trial of the ChAdOx1 nCOV-19 vaccine asked participants to provide a weekly self-administered nose and throat swab starting 1 week after administration of the first vaccine (or placebo). This study revealed that among those who were infected, vaccinated individuals had lower peak viral load and shorter duration of RT-PCR^+^ results for SARS-CoV-2 compared with controls, suggesting that the vaccine is effective in reducing transmission (25).

Interim results of Gam-COVID-Vac (Sputnik V) showed that vaccine efficacy, defined as a decrease in RT-PCR–confirmed symptomatic infection, 21 days after the first dose was 91.6% (95% CI, 85.6%–95.2%), with similar efficacy, 91.1% (95% CI, 83.8%–95.1%) 7 days after the second dose (26). Efficacy was similar in those above 60 years of age. All moderate or severe cases of COVID-19 occurred in the placebo group.

A phase II trial of a single dose of the CanSino vaccine showed seroconversion rates of 96%–97% for antibodies against RBD and T cell responses in 88%–90% (27). Only anecdotal results are available from the single-dose phase III trial of CanSino Biologics vaccine, which involved 30,000 participants, with news reports indicating an efficacy of 65.7% in preventing symptomatic cases and 91% efficacy in preventing severe disease. The CanSino vaccine has been used for vaccinating the military in China and received EUA in several other countries.

To date, efficacy of all viral vector vaccines in preventing symptomatic SARS-CoV-2 infection exceeded the prespecified goal of 50%. Viral vector vaccines are likely to be easier to distribute and administer than mRNA vaccines. The lack of a need for deep freezing and demonstrated efficacy of single-dose regimens (for Ad26.COV2.S and CanSino) have the potential to greatly simplify the logistical challenges of mass vaccination.

### Protein subunit.

Multiple vaccines with excellent efficacy and safety records use recombinant protein combined with an adjuvant, such as hepatitis B and zoster vaccines. This vaccine technology brings a long record of real-world effectiveness and safety along with comparatively simple needs for storage, transportation, and administration.

Novavax NVX-CoV2373 is a protein subunit SARS-CoV-2 vaccine consisting of nanoparticles containing baculovirus-expressed full-length SARS-CoV-2 S protein produced in insect cells and Matrix-M1, a saponin-based adjuvant. Preliminary results of a phase III trial were provided in a press release. In the United Kingdom, efficacy was 89.3% (95% CI, 75.2%–95.4%) against any symptomatic infection 7 days after the second dose. Post hoc analysis revealed an efficacy of 95.6% against the original SARS-CoV-2 strain and 89.6% against the B.1.1.7 variant, then prevalent in the United Kingdom. Limited data from a phase IIb trial in South Africa showed lower efficacy of 60% (95% CI, 19.9%–80.1%), with 25 of 27 viruses sequenced from COVID-19 cases found to be the B.1.351 variant, prevalent in South Africa.

### Inactivated virus.

Several candidate SARS-CoV-2 vaccines employ this well-established method in which reference strains of the targeted virus are grown and then inactivated (most often with heat, formalin, or β-propiolactone). Results of phase III trials of vaccines using this platform have not been officially released at this time.

Phase I/II trials of the CoronaVac vaccine (Sinovac) suggested that efficacy may be lower in individuals older than 60 years. Only anecdotal preliminary results of phase III trials conducted in various countries have been reported on, but not provided for review, with efficacy in Turkey reported as 91.3% based on 29 cases and 65.3% in Indonesia. Efficacy in Brazil was initially reported as 78% but later revised to 50% after additional data were included, though efficacy in preventing severe infection was higher. Only news reports are available of the preliminary results from the Sinopharm vaccine, showing 79% efficacy; however, the United Arab Emirates announced that this vaccine was 86% effective, according to interim results of its phase III trial.

Both Sinovac and Sinopharm vaccines have been approved for EUA in China and also in some other countries. Notably, the Merck SARS-CoV-2 vaccines with this technology were abandoned due to disappointing immunogenicity results.

## Durability of protection

Currently reported efficacy estimates are based on data during the first 3–4 months after the first dose of candidate vaccines. Longer-term follow-up data from the phase III trials are needed to determine durability of protection; however, these data may be confounded by unblinding of participants who have access to EUA vaccines and potential cross-over of placebo group to vaccine group. Continued follow-up of these participants will still be informative to monitor any future infection with SARS-CoV-2 and possible immunological surrogates for protection. Data from the Oxford University Hospitals health care workers support an inverse association between baseline anti-S and anti-nucleocapsid antibody titers and incidence of SARS-CoV-2 infection during a follow-up period of 31 weeks (7). Whether the durability of protection after vaccination is similar to that after natural infection is unclear. It will also be important to determine if the rate of decline in antibody titers is influenced by age, sex, race/ethnicity, and/or medical comorbidities. In addition to antibody response, CD4^+^ and CD8^+^ T cell responses have been detected in vaccine recipients (28–30), and long-term follow-up will clarify the role of cellular immunity in durability of protection.

## Efficacy of vaccines against emerging SARS-CoV-2 variants

The emergence of strains and variants of SARS-CoV-2 has already been observed (31). Some of these variants may escape neutralization by existing vaccines that present the S protein of the original (Wuhan) strain (32). The first prevalent variant, D614G, was reported in April 2020. It is located in the S protein and has been shown to increase replication and transmissibility but does not escape recognition by neutralizing antibodies. Since then, multiple variants with mutations in the S protein have been reported, and there is emerging data on how these mutations affect transmissibility and escape from immune response (33). This raises concerns about efficacy of current SARS-CoV-2 vaccines. The two variants that have received the most attention are B.1.1.7 and B.1.351.

In vitro studies found that neutralizing activity of sera from recipients of the mRNA vaccines BNT162b2 and mRNA-1273 against the S protein of the B.1.1.7 variant is only approximately 2-fold lower than the neutralizing activity against the S protein of the ancestral (Wuhan) strain (34–36). Similar results were observed for the protein subunit NVX-CoV2373 vaccine (37). By contrast, neutralizing activity of sera from recipients of the BNT162b2 and mRNA-1273 vaccines against the S protein of the B.1.351 variant is 6.5- to 8.6-fold lower compared with the ancestral strain (38, 39). These studies also showed that the triple mutations (K417N/E484K/N501Y) as well as the E484K mutation alone in the RBD lead to a more than 3-fold decrease in neutralizing activity. These findings have implications for the P.1 or B.1.128 variant, which also contains K417N/E484K/N501Y mutations, and the P.2 variant, which contains the E484K but not the N501Y mutation, which are rapidly increasing in Brazil.

Data on in vivo efficacy of SARS-CoV-2 vaccines against the B.1.1.7 and the B.1.351 variants are limited. Data from a phase II/III study of ChAdOx1 nCoV19 vaccine in the United Kingdom showed that virus neutralization activity by vaccine-induced antibodies was 9-fold lower against the B.1.1.7 variant compared with a canonical non-B.1.1.7 lineage; however, vaccine efficacy against symptomatic RT-PCR–confirmed infection was similar, 74.6% (95% CI, 41.6–88.9%) and 84% (95% CI, 70.7–91.4%), respectively (25). Results of a study in South Africa including 1467 seronegative adults showed that the ChAdOx1 nCoV19 vaccine had an efficacy against mild-to-moderate infection more than 14 days after the second dose of only 21.9% (95% CI, 49.9%–59.8%) and an efficacy against the B.1.351 variant of only 10.4% (95% CI, 76.8%–54.8%) (40).

The Ad26.COV2.S vaccine phase III trial included participants in Brazil and South Africa when the P.2 and B.1.351 variants (with concerns for immune evasion) were becoming dominant. Of the confirmed cases sequenced, the B.1.351 variant was present in 94.5% cases in South Africa and none in the US or Brazil, while the P.2 variant was present in 69.4% cases in Brazil, 2.2% in South Africa, and 1% in the US. Despite the presence of these variants, the efficacy of the Ad26.COV2.S vaccine exceeded the prespecified goal of 50% in preventing symptomatic infections (72% in the US, 68.1% in Brazil, and 64% in South Africa).

Preliminary data of the NVX-CoV2373 vaccine, based on press release, showed that overall efficacy in the phase III trial was 89.3%. Based on analysis of 62 cases, efficacy was calculated to be 95.6% against the original strain and 85.6% against the B.1.1.7 variant. By contrast, results of a phase IIb trial in South Africa, showed the overall efficacy was lower, 60% (95% CI, 19.9%–80.1%), and preliminary sequencing data found that 92.6% of cases were due to the B.1.351 variant.

The phase III trials of the two mRNA vaccines, BNT162b2 and mRNA-1273, were conducted before the emergence of the B.1.1.7, P.1, P.2, and B.1.351 variants. Data from in vitro studies suggest that clinical efficacy of these vaccines against the B.1.1.7 variant may still be preserved due to the so-called “cushion” effect of the high-titer neutralizing antibodies in the vaccine recipients. This is supported by greater than 90% effectiveness of BNT162b2 in preventing documented infection and symptomatic infection in the mass vaccination program in Israel, during a time when up to 80% of SARS-CoV-2 isolates in Israel were of the B.1.1.7 variant (41). However, efficacy against the B.1.351 and possibly the P.1 or P.2 variant may be reduced in those with low antibody titers.

The foregoing highlights the importance of surveillance for emerging variants and long-term follow-up of vaccine recipients. They also raise concerns about the need for revaccination at regular intervals to protect against vaccine escape variants.

## Real-world effectiveness of SARS-CoV-2 vaccines

The phase III trials of SARS-CoV-2 vaccines were not designed to demonstrate their ability to reduce the risk of transmission or severe disease. Studies of real-world effectiveness are providing us with our first compelling evidence.

By February 1, 2021, a sufficient percentage of the population of Israel had received the BNT162b2 vaccine, allowing for some estimates of the real-world effectiveness of the vaccine. Estimated vaccine effectiveness based on nearly 600,000 vaccinated persons and the same number of unvaccinated persons at 7 or more days after the second dose was 92% (95% CI, 88%–95%) for documented infection, 94% (95% CI, 87%–98%) for symptomatic disease, 87% (95% CI, 55%–100%) for hospitalization, and 92% (95% CI, 75%–100%) for severe disease. Estimation of the effectiveness in preventing death was limited by the small number of events and was reported as 72% (95% CI, 19%–100%) for the period from days 14 through 20 after the first dose. Based on publicly available data, geographic regions vaccinated earlier had a greater decline in COVID-19 cases as compared with those vaccinated later (42). Similar findings have been reported from Scotland, where an 85% (95% CI, 76%–91%) or 94% (95% CI, 73%–99%) reduction in COVID-19 hospitalizations was observed 28–34 days after the first dose of BNT162b2 or ChAdOx1 nCoV19 vaccines, respectively (43). Together, these results are consistent with the phase III trial findings and further support the effectiveness of the SARS-CoV-2 vaccines against severe COVID-19.

## Safety

**Figure 3:**
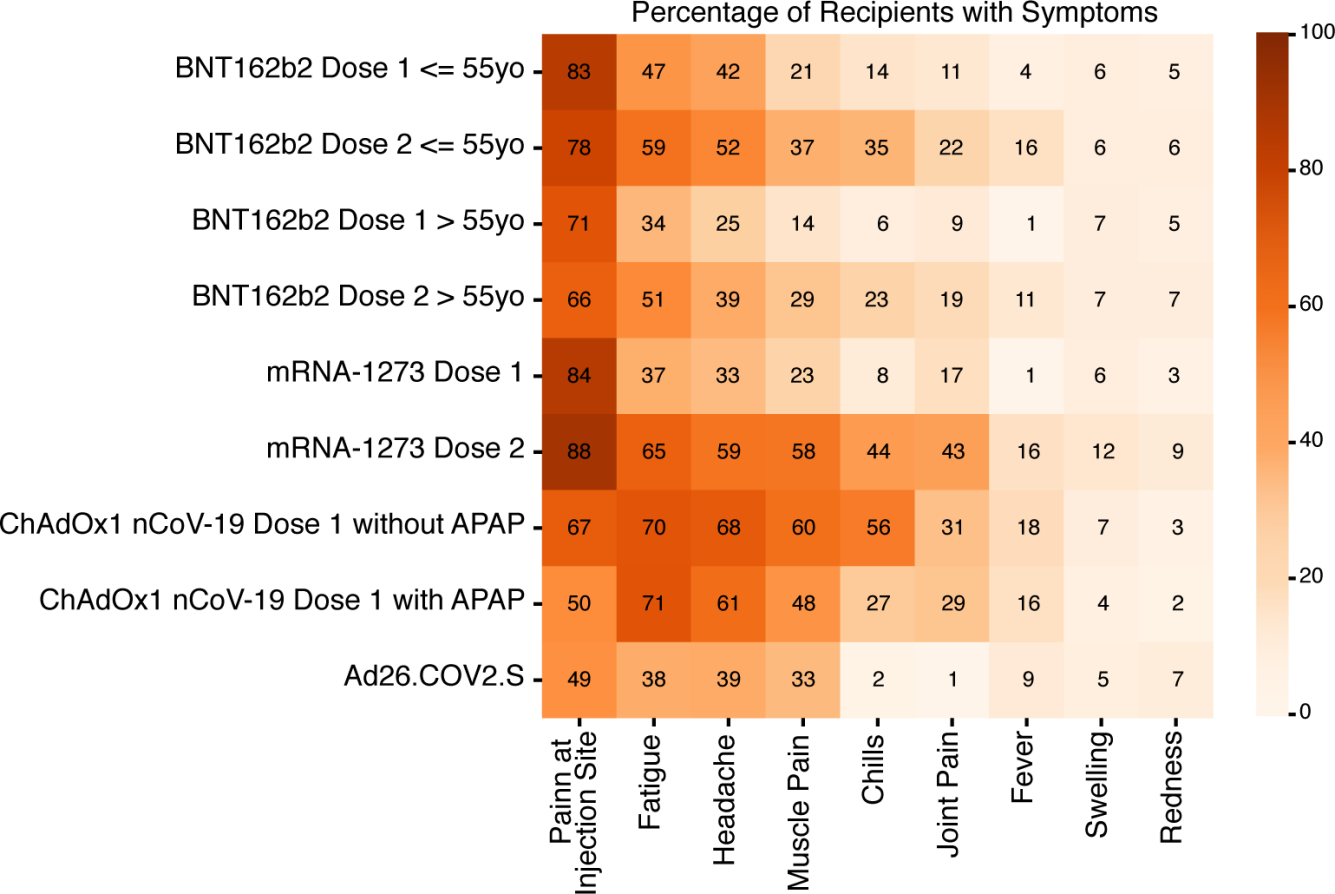
Frequency of common adverse events after vaccination, as reported in the interim analyses of the phase III trials. All events are solicited adverse events, aside from chills and joint pain for Ad26.COV2.S, which were nonsolicited. APAP, acetaminophen. Illustrated by Rachel Davidowitz.

All SARS-CoV-2 vaccines have a similar safety profile, with 60%–80% of participants in phase III trials experiencing local (injection site pain, redness, or swelling) reactions and 30%–60% experiencing systemic (fever, chills, headache, fatigue, muscle pain, and joint pain) adverse events, but the events have been generally transient and mild (Figure 3). Available data suggest that reactions are more frequent and severe after the second dose compared with the first dose and milder in older persons. Safety of the two mRNA vaccines have been confirmed in an analysis of the first month of safety monitoring after nearly 14 million doses had been administered in the US (44). There is emerging evidence that those with confirmed history of prior infection with SARS-CoV-2 may be more likely to have side effects from the vaccines and to have higher antibody titers after the first vaccine dose, suggesting an anamnestic (or enhanced) immune response (45–47).

People with a known or suspected allergy or history of anaphylaxis or other serious adverse reactions to vaccines or their excipients were excluded from the clinical trials. Early safety monitoring after EUA of the two mRNA vaccines detected 21 cases of anaphylaxis after administration of BNT162b2 vaccine (11.1 cases per million first doses) and 10 cases after mRNA-1273 vaccine (2.5 per million first doses) (48). A majority (80%–90%) of these cases occurred in those with a history of allergies or allergic reactions, with more than 70% manifesting symptoms within 30 minutes of vaccine receipt. The significance of the very rare observed incidence of anaphylaxis remains unclear, as does the possible underlying etiology (49).

Long-term safety of all the vaccines will continue to be monitored during the planned 2-year follow-up. In addition to the long-established passive surveillance system — Vaccine Adverse Event Reporting System — the CDC has set up an active surveillance system (v-safe) to track safety as well as symptoms and diagnoses of COVID-19 after receipt of EUA SARS-CoV-2 vaccines. One concern is the potential for antibody-dependent enhancement of infection in vaccine recipients if they are exposed to SARS-CoV-2, though this has not been documented at this time.

The safety of SARS-CoV-2 vaccines in populations not included in phase III trials remains to be determined, notably children and adolescents, pregnant or lactating women, immunocompromised persons, persons with unstable medical comorbidities (persons with stable comorbidities were allowed), and persons with history of allergies.

## Path from vaccines to control of SARS-CoV-2

The remarkable speed in developing the SARS-CoV-2 vaccines and the high efficacy in phase III trials are a tribute to collaborative science, success of the novel platforms, enthusiasm of both investigators and participants, and financial support from governments. It has been estimated that 70%–90% of the population must be vaccinated to achieve herd immunity. However, herd immunity is dependent on efficacy of the vaccines used and durability of vaccine-induced immunity as well as natural immunity; and infection rates are linked to continued safe practices, including social distancing and masking. Several barriers in the path from vaccine development to widespread vaccination must be overcome, including adequate supplies to distribute vaccines where they are needed and public acceptance of vaccination.

Companies involved in the development of SARS-CoV-2 vaccines have received preapproval orders and have manufactured millions of doses before completion of the phase III trials. Nonetheless, supply lags behind the demand. Distribution has been hampered by the need for some of the vaccines to be kept at –20°C and, in the case of BNT162b, at –70°C. These stringent requirements make it difficult for the mRNA vaccines to be deployed. The need for two doses of vaccine also greatly increases the complexity of mass vaccination at a time when health departments struggle with getting the first dose of vaccines to those in need. Having the Ad26.COV2.S vaccine with high efficacy after a single dose may alleviate this pressure.

Another problem with distribution is equity. While it is generally accepted that those at increased risk for exposure to SARS-CoV-2 (e.g., healthcare workers) or for a severe course of COVID-19 (e.g., elderly individuals) should be prioritized, identifying high-risk individuals and reaching them can be challenging. After the first 2–3 weeks, it became apparent that the tier-approach based on individual risk factors adopted in the US was too complex, prompting a shift to focus on those aged 65 years and older. However, in the US, there continues to be a mismatch between the number of doses distributed and the number of high-risk people in many cities and counties. In addition, the requirement for digital appointments, the need to travel to a vaccination site, and the wait in line for hours at some of these sites have magnified the health disparities laid bare by the COVID-19 pandemic. In contrast, the mass-vaccination programs in countries with integrated single-payer healthcare systems, such as Israel (50) and the United Kingdom (43), accomplished mass vaccination earlier and more comprehensively. The intersection between healthcare systems and the pace of mass vaccination programs is a crucial area to study in the aftermath of the COVID-19 pandemic.

Even when all the logistics work smoothly, vaccine hesitancy remains a challenge. Concerns about side effects, a lack of trust in the process, and a desire to wait for the “best” vaccine are driving factors for the hesitancy. We support each individual choosing the vaccine that is the best fit for his or her needs, but we urge caution in directly comparing the reported efficacy across SARS-CoV-2 vaccine studies. Differences in the specific study participants, timing and location of studies, and infection rates and prevalence of SARS-CoV-2 variants in the community at the time of the trials all could contribute to the observed efficacy in ways difficult to correct for. The key point to note is that all the vaccines approved for emergency use have surpassed their prespecified objective of at least 50% efficacy. In both randomized phase III trials and emerging real-world effectiveness data, the vaccines that have received EUA have excellent safety and efficacy profiles. Further, vaccination is a public health intervention. How many people are vaccinated and how quickly vaccination can be accomplished are likely far stronger determinants of real-word effectiveness than the specific vaccine in use. For an individual, the best protection for themselves and their community is to accept *any* EUA vaccine as soon as it is offered. A comprehensive plan that incorporates multiple strategies tailored to reasons for hesitancy and logistical barriers in each population along with simple and consistent messages about safety and efficacy will be required to increase vaccine adoption.

Science has enabled us to rapidly develop vaccines against SARS-CoV-2 and to demonstrate their safety and efficacy. It is now time for humanity to help us cross the gap from vaccine to vaccination.

## Author contributions

JG contributed to the literature review, data synthesis, initial draft, figure preparation, and manuscript editing. NL contributed to figure preparation and manuscript editing. A.S. Lauring contributed to literature review and manuscript editing. A.S. Lok contributed to literature review, data synthesis, manuscript drafting, and editing. All authors provided final approval.

## Figures and Tables

**Table 1:**
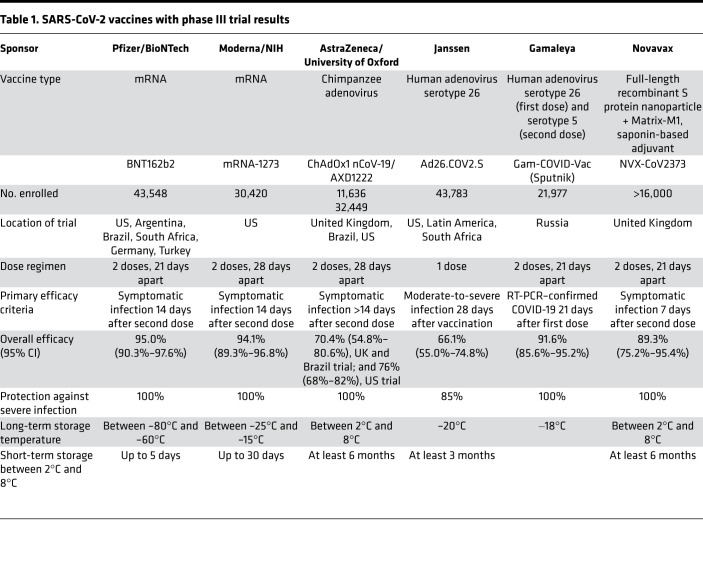
SARS-CoV-2 vaccines with phase III trial results

## Version 1 04/06/2021

In-Press Preview

## Version 2 05/10/2021

Electronic publication

## References

[1] Carfì A (2020). Persistent symptoms in patients after acute COVID-19. JAMA.

[2] Chopra V, et al. Sixty-day outcomes among patients hospitalized with COVID-19 [published online November 11, 2020]. Ann Intern Med . 10.7326/m20-5661 PMC7707210 33175566

[3] World Health Organization. Draft landscape and tracker of COVID-19 candidate vaccines. https://www.who.int/publications/m/item/draft-landscape-of-covid-19-candidate-vaccines Updated April 2, 2021. Accessed March 18, 2021

[4] Gudbjartsson DF (2020). Spread of SARS-CoV-2 in the Icelandic population. N Engl J Med.

[5] Stephens DS, McElrath MJ (2020). COVID-19 and the path to immunity. JAMA.

[6] Sekine T (2020). Robust T cell immunity in convalescent individuals with asymptomatic or mild COVID-19. Cell.

[7] Lumley SF (2021). Antibody status and incidence of SARS-CoV-2 infection in health care workers. N Engl J Med.

[8] Blanco-Melo D (2020). Imbalanced host response to SARS-CoV-2 drives development of COVID-19. Cell.

[9] Lokugamage KG (2020). Type I interferon susceptibility distinguishes SARS-CoV-2 from SARS-CoV. J Virol.

[10] Shuai H (2020). Differential immune activation profile of SARS-CoV-2 and SARS-CoV infection in human lung and intestinal cells: Implications for treatment with IFN-β and IFN inducer. J Infect.

[11] Lazear HM (2019). Shared and distinct functions of type I and type III interferons. Immunity.

[12] Mercado NB (2020). Single-shot Ad26 vaccine protects against SARS-CoV-2 in rhesus macaques. Nature.

[13] van Doremalen N (2020). ChAdOx1 nCoV-19 vaccine prevents SARS-CoV-2 pneumonia in rhesus macaques. Nature.

[14] Corbett KS (2020). Evaluation of the mRNA-1273 vaccine against SARS-CoV-2 in nonhuman primates. N Engl J Med.

[15] Heil F (2004). Species-specific recognition of single-stranded RNA via Toll-like receptor 7 and 8. Science.

[16] Buchbinder SP (2008). Efficacy assessment of a cell-mediated immunity HIV-1 vaccine (the Step Study): a double-blind, randomised, placebo-controlled, test-of-concept trial. Lancet Lond Engl.

[17] Polack FP (2020). Safety and efficacy of the BNT162b2 mRNA Covid-19 vaccine. N Engl J Med.

[18] Baden LR (2021). Efficacy and safety of the mRNA-1273 SARS-CoV-2 vaccine. N Engl J Med.

[19] Pollard AJ (2021). Safety and immunogenicity of a two-dose heterologous Ad26.ZEBOV and MVA-BN-Filo Ebola vaccine regimen in adults in Europe (EBOVAC2): a randomised, observer-blind, participant-blind, placebo-controlled, phase 2 trial. Lancet Infect Dis.

[20] Bos R (2020). Ad26 vector-based COVID-19 vaccine encoding a prefusion-stabilized SARS-CoV-2 Spike immunogen induces potent humoral and cellular immune responses. NPJ Vaccines.

[21] Folegatti PM (2020). Safety and immunogenicity of the ChAdOx1 nCoV-19 vaccine against SARS-CoV-2: a preliminary report of a phase 1/2, single-blind, randomised controlled trial. Lancet Lond Engl.

[22] Sadoff J, et al. Safety and efficacy of single-dose Ad26.COV2.S vaccine against Covid-19 [published online April 21, 2021]. N Engl J Med . 10.1056/NEJMoa2101544 PMC8220996 33882225

[23] Voysey M (2021). Safety and efficacy of the ChAdOx1 nCoV-19 vaccine (AZD1222) against SARS-CoV-2: an interim analysis of four randomised controlled trials in Brazil, South Africa, and the UK. Lancet Lond Engl.

[24] Voysey M (2021). Single-dose administration and the influence of the timing of the booster dose on immunogenicity and efficacy of ChAdOx1 nCoV-19 (AZD1222) vaccine: a pooled analysis of four randomised trials. Lancet Lond Engl.

[25] Emary KRW, et al. Efficacy of ChAdOx1 nCoV-19 (AZD1222) vaccine against SARS-CoV-2 VOC 202012/01 (B.1.1.7) [preprint]. 10.2139/ssrn.3779160 Posted on SSRN February 4, 2021

[26] Logunov DY (2021). Safety and efficacy of an rAd26 and rAd5 vector-based heterologous prime-boost COVID-19 vaccine: an interim analysis of a randomised controlled phase 3 trial in Russia. Lancet Lond Engl.

[27] Zhu FC (2020). Immunogenicity and safety of a recombinant adenovirus type-5-vectored COVID-19 vaccine in healthy adults aged 18 years or older: a randomised, double-blind, placebo-controlled, phase 2 trial. Lancet Lond Engl.

[28] Jackson LA (2020). An mRNA vaccine against SARS-CoV-2 - preliminary report. N Engl J Med.

[29] Ramasamy MN (2021). Safety and immunogenicity of ChAdOx1 nCoV-19 vaccine administered in a prime-boost regimen in young and old adults (COV002): a single-blind, randomised, controlled, phase 2/3 trial. Lancet Lond Engl.

[30] Stephenson KE, et al. Immunogenicity of the Ad26.COV2.S vaccine for COVID-19 [published online March 11, 2021]. JAMA . 10.1001/jama.2021.3645 PMC7953339 33704352

[31] Lauring AS, Hodcroft EB (2021). Genetic variants of SARS-CoV-2-what do they mean?. JAMA.

[32] Li Q, et al. SARS-CoV-2 501Y.V2 variants lack higher infectivity but do have immune escape [published by February 23, 2021]. Cell . 10.1016/j.cell.2021.02.042﻿﻿ PMC7901273 33735608

[33] McCarthy KR (2021). Recurrent deletions in the SARS-CoV-2 spike glycoprotein drive antibody escape. Science.

[34] Liu Y, et al. Neutralizing activity of BNT162b2-elicited serum — preliminary report [published online February 17, 2021]. N Engl J Med . 10.1056/nejmc2102017 PMC7944950 33684280

[35] Wu K, et al. Serum neutralizing activity elicited by mRNA-1273 vaccine — preliminary report [published online February 17, 2021]. N Engl J Med . 10.1056/nejmc2102179 PMC8008744 33730471

[36] Wang Z, et al. mRNA vaccine-elicited antibodies to SARS-CoV-2 and circulating variants [preprint]. 10.1101/2021.01.15.426911 Posted on bioRxiv February 10, 2021 PMC8503938 33567448

[37] Shen X, et al. SARS-CoV-2 variant B.1.1.7 is susceptible to neutralizing antibodies elicited by ancestral Spike vaccines [preprint]. 10.1101/2021.01.27.428516 Posted on bioRxiv January 23, 2021 PMC7934674 33705729

[38] Wu K, et al. mRNA-1273 vaccine induces neutralizing antibodies against spike mutants from global SARS-CoV-2 variants [preprint]. 10.1101/2021.01.25.427948 Posted on bioRxiv January 25, 2021

[39] Wang P, et al. Antibody resistance of SARS-CoV-2 variants B.1.351 and B.1.1.7 [preprint]. 10.1101/2021.01.25.428137 Posted on bioRxiv February 12, 2021 33684923

[40] Madhi SA, et al. Efficacy of the ChAdOx1 nCoV-19 Covid-19 vaccine against the B.1.351 Variant [published online March 16, 2021]. N Engl J Med . 10.1056/nejmoa2102214 PMC7993410 33725432

[41] Dagan N, et al. BNT162b2 mRNA Covid-19 vaccine in a nationwide mass vaccination setting [published online February 24, 2021]. N Engl J Med . 10.1056/nejmoa2101765 PMC7944975 33626250

[42] Rossman H, et al. Patterns of COVID-19 pandemic dynamics following deployment of a broad national immunization program [preprint]. 10.1101/2021.02.08.21251325 Posted on medRxiv March 8, 2021

[43] Vasileiou E. Effectiveness of first dose of COVID-19 vaccines against hospital admissions in Scotland: national prospective cohort study of 5.4 Million people [preprint]. 10.2139/ssrn.3789264 Posted on SSRN February 19, 2021

[44] Gee J (2021). First month of COVID-19 vaccine safety monitoring — United States, December 14, 2020–January 13, 2021. MMWR.

[45] Saadat S, et al. *Single Dose Vaccination in Healthcare Workers Previously Infected with SARS-CoV-2 [Internet*]. Infectious Diseases (except HIV/AIDS); 2021:

[46] Krammer F, et al. Robust spike antibody responses and increased reactogenicity in seropositive individuals after a single dose of SARS-CoV-2 mRNA vaccine [preprint]. 10.1101/2021.01.29.21250653 Posted on medRxiv February 1, 2021

[47] Ciccone EJ, et al. SARS-CoV-2 seropositivity after infection and antibody response to mRNA-based vaccination [preprint]. 10.1101/2021.02.09.21251319 Posted on medRxiv February 22, 2021

[48] CDC COVID-19 Response Team, Food And Drug Administration (2021). Allergic reactions including anaphylaxis after receipt of the first dose of Moderna COVID-19 vaccine — United States, December 21, 2020-January 10, 2021. MMWR.

[49] Castells MC, Phillips EJ (2021). Maintaining safety with SARS-CoV-2 Vaccines. N Engl J Med.

[50] Aran D. Estimating real-world COVID-19 vaccine effectiveness in Israel using aggregated counts [preprint]. 10.1101/2021.02.05.21251139 Posted on medRxiv February 19, 2021

[51] Clopper C, Pearson ES (1934). The use of confidence or fiducial limits illustrated in the case of the binomial. Biometrika.

